# Antiviral Potential of Nanoparticles—Can Nanoparticles Fight Against Coronaviruses?

**DOI:** 10.3390/nano10091645

**Published:** 2020-08-21

**Authors:** Sangiliyandi Gurunathan, Muhammad Qasim, Youngsok Choi, Jeong Tae Do, Chankyu Park, Kwonho Hong, Jin-Hoi Kim, Hyuk Song

**Affiliations:** 1 Department of Stem Cell and Regenerative Biotechnology, Konkuk University, Seoul 05029, Korea; sangiliyandi@konkuk.ac.kr (S.G.); choiys3969@konkuk.ac.kr (Y.C.); dojt@konkuk.ac.kr (J.T.D.); chankyu@konkuk.ac.kr (C.P.); hongk@konkuk.ac.kr (K.H.); jhkim541@konkuk.ac.kr (J.-H.K.); 2 Center of Bioengineering and Nanomedicine, Department of Food Science, University of Otago, Dunedin 9054, New Zealand; muhammadqasim.qasim@otago.ac.nz

**Keywords:** antiviral agent, nanoparticle, coronavirus, viral mechanism of entry, antiviral mechanism, therapeutic approaches, SARS-CoV-2, COVID-19

## Abstract

Infectious diseases account for more than 20% of global mortality and viruses are responsible for about one-third of these deaths. Highly infectious viral diseases such as severe acute respiratory (SARS), Middle East respiratory syndrome (MERS) and coronavirus disease (COVID-19) are emerging more frequently and their worldwide spread poses a serious threat to human health and the global economy. The current COVID-19 pandemic, caused by severe acute respiratory syndrome coronavirus 2 (SARS-CoV-2). As of 27 July 2020, SARS-CoV-2 has infected over 16 million people and led to the death of more than 652,434 individuals as on 27 July 2020 while also causing significant economic losses. To date, there are no vaccines or specific antiviral drugs to prevent or treat COVID-19. Hence, it is necessary to accelerate the development of antiviral drugs and vaccines to help mitigate this pandemic. Non-Conventional antiviral agents must also be considered and exploited. In this regard, nanoparticles can be used as antiviral agents for the treatment of various viral infections. The use of nanoparticles provides an interesting opportunity for the development of novel antiviral therapies with a low probability of developing drug resistance compared to conventional chemical-based antiviral therapies. In this review, we first discuss viral mechanisms of entry into host cells and then we detail the major and important types of nanomaterials that could be used as antiviral agents. These nanomaterials include silver, gold, quantum dots, organic nanoparticles, liposomes, dendrimers and polymers. Further, we consider antiviral mechanisms, the effects of nanoparticles on coronaviruses and therapeutic approaches of nanoparticles. Finally, we provide our perspective on the future of nanoparticles in the fight against viral infections.

## 1. Introduction

Infectious diseases are caused by pathogenic microorganisms that spread directly or indirectly from one person to another [1]. Zoonotic diseases are infectious diseases of animals that can cause disease in humans when the causal agent is transmitted from animal to human; the diseases can account for hundreds of thousands of deaths worldwide. Infectious diseases pose a significant threat to both human health and the global economy; to date, we know of about 200 infectious diseases. Fortunately, only a handful of these diseases are responsible for significant morbidity and mortality [2,3]. Among them, human immunodeficiency virus/acquired immune deficiency syndrome (HIV/AIDS), tuberculosis and malaria are the most pronounced. In addition, several outbreaks of infectious diseases have occurred recently including Ebola, Zika and avian influenza as well as the coronavirus (CoV) diseases severe acute respiratory syndrome (SARS; caused by SARS-CoV), Middle East Respiratory Syndrome (MERS; caused by MERS-coronavirus (MERS-CoV)) and COVID-19 (caused by SARS coronavirus 2 (SARS-CoV-2). These diseases originated in West Africa, South America, Asia, Asia, the Middle East and Asia, respectively, before spreading to other parts of the world [4,5,6,7,8]. In addition several other viral diseases also found to be widely spread such as Hantavirus which is transmitted by rodents, Chikungunya and Rift Valley fever virus (RVFV) virus by mosquitos, paramyxoviruses such as rubulaviruses, Nipah and Hendra viruses from bats and so forth. Infectious diseases account for ~20% of global mortality and viruses are responsible for about one-third of these deaths [1]. For example, SARS-CoV-2, the causal virus of coronavirus disease (COVID-19), is transmitted directly from one human to another. The outbreak of COVID-19 began in late 2019 and as of 27 July 2020, SARS-CoV-2 has infected more than 16,430,566 individuals and led to the death of more than 652,434 individuals in 215 countries around the world (https://www.worldometers.info/coronavirus/accessed on 27 July 2020). The number of infected cases and deaths are still seriously increasing every day, affecting essentially every country worldwide.

Coronaviruses (CoVs) are a large family of RNA viruses. They are the major pathogen of emerging respiratory disease outbreaks and can cause a variety of diseases in mammals and birds, from which they can be isolated [9]. CoVs primarily infect the upper respiratory and gastrointestinal tract of mammals and birds. The major symptoms of CoV infection of the upper respiratory tract in humans include cough, fever, and, in more severe cases, difficulty in breathing has been reported with potential fatality from SARS-CoV, MERS-CoV and SARS-CoV-2. The clinical symptoms of COVID-19, including acute respiratory disorder induced by either highly homogenous SARS-CoV-2 or other secondary pathogens, suggest that excessive inflammation, oxidation and an exaggerated immune response very likely contribute to COVID-19 pathology. This initial response can lead to a cytokine storm and subsequent progression to acute lung injury/acute respiratory distress syndrome and often death [10]. SARS-CoV-2 binds to human angiotensin-converting enzyme-2 (ACE2) receptors and not only induces pneumonia but also it induces multisystem illness with involvement of different organs and potential for systemic complications [11]. Increasing evidence suggest that SARS-CoV-2 induce significant abnormalities compatible with hypercoagulability with hyperfibrinogenemia and clinically a high prevalence of thromboembolic events. Further it causes potential for large vessel thrombosis and major thromboembolic sequelae including pulmonary embolism (PE) deep vein thrombosis (DVT) and thrombosis in extracorporeal circuits and arterial thrombosis [12,13,14]. COVID-19 is closely related to SARS, which swept the world in 2002 and 2003. SARS-CoV infected about 8000 people and killed about 800. MERS outbreaks have occurred sporadically since 2012, infecting about 2500 people and resulting in nearly 900 deaths. COVID-19 is different from other two CoVs resulting in more severe effects and spreading faster in humans. COVID-19 has become a pandemic with millions of infected patients over a period of less than one year since its suspected outbreak. These alarming disease statistics serve to emphasize the global concern over infectious diseases and the enormous influence on the global socio-economic and health-care sectors. Although we may develop a drug for a particular viral infection, viral mutations may lead to drug resistance, rendering it ineffective. This was observed in the case of HIV and influenza treatment. However, mixture of antiviral agents such as HIV2 nucleoside reverse transcriptase inhibitors (NRTIs) plus an integrase strand transfer inhibitor (InSTI) and also other effective regimens include nonnucleoside reverse transcriptase inhibitors or boosted protease inhibitors with 2 NRTIs showed promising effect on HIV patients. These antiretroviral drugs (ARVs) can sustain HIV suppression and can prevent new HIV infection. Hence, drug resistance is a public health threat, eventually increasing morbidity and mortality [15,16,17]. 

The best approach to preventing viral infections is vaccination; however, the development of vaccines is time consuming, expensive and requires sophisticated equipment and lengthy protocols. A limited number of vaccines are available for infectious diseases but the ones that exist are not always equally available worldwide. Some currently available broad-spectrum antivirals including lopinavir/ritonavir, neuraminidase inhibitors, EK1 peptide, RNA synthesis inhibitors, nucleoside analogs and HIV-protease inhibitors could be effective, alternative medicines for COVID-19 [18]. Further, interferons (IFNs) seem to be partially effective against CoVs; a combination of IFNs and ribavirin exhibited increased inhibitory activity in vitro when compared to IFNs alone against some CoVs [19,20]. The drug EIDD-2801, used for pandemic influenza viral infections, is another alternative worthy of consideration for the treatment of COVID-19 [21].

An in vitro study suggested that the antiviral drug remdesivir and the anti-malaria drug chloroquine can potentially control COVID-19. Chloroquine has been used to treat malaria for many years; however, the mechanism of action of chloroquine against viral infections remains elusive [22]. Liu et al. performed the comparative analysis between hydroxychloroquine and chloroquine against SARS-CoV-2 infected patients [23]. The results shows that hydroxychloroquine is less toxic and more effective to inhibit SARS-CoV-2 infection. Corticosteroid ciclesonide blocks coronavirus RNA replication by targeting viral NSP15. Conversely, corticosteroid did not suppress replication of respiratory syncytial virus or influenza virus [24]. Bioinformatics analysis revealed that both esterified and non-esterified derivatives of ciclesonide had the capacity to interact with NSP-15, thereby possessing the capacity to inhibit replication of the SARS-CoV-2 viral genome [25]. Ivermectin, an FDA-approved anti-parasitic drug inhibits RNA replication and also decrease many fold RNA virus load [26]. Ivermectin plays significant role in several biological mechanisms and it could be potential antiviral agents against various type of viruses including SARS-CoV-2 [27]. Ianevski et al. (2020) adopted a screening strategy to find suitable drug against SARS-C0V-2 by neutralization assay using sera from various SARS-CoV-2 infected patients [28]. They found that the most potent sera from recovered patients for the treatment of SARS-CoV-2-infected patients. They found that a combination of orally available virus directed nelfinavir and host-directed amodiaquine exhibited the highest synergy against SARS-CoV-2. Wang et al. reported that anti-influenza drug called arbidol efficiently inhibited SARS-CoV-2 infection by the mechanism of blocking of entry of virus into the host cells by impeding viral attachment [29]. Remdesivir (GS-5734) shows broad spectrum antiviral activity against several RNA viruses by interfering with RNA-dependent RNA polymerase (RdRp, also called NSP12 polymerase), even in the presence of an exoribonuclease (ExoN) with proof-reading activity, as demonstrated in an in vitro cell line and mouse model [30]. Remdesivir also showed positive results when tested in a rhesus macaque model of MERS-CoV infection [31,32]. Therefore, the drug could be effective for both the prevention and treatment of human CoV (HCoV) infections. Holshue et al. (2020) reported the first case of a COVID-19 patient being treated with remdesivir in the United States [33].

Protease inhibitors such as lopinavir and ritonavir have been successfully used to treat HIV infection and they have improved the outcomes of MERS-CoV and SARS-CoV patients [34,35,36]. Recent studies reported that administration of lopinavir/ritonavir (Kaletra^®^, AbbVie, North Chicago, IL, USA) significantly reduced β-CoV titers of a COVID-19 patient in Korea [37]. The combination of Chinese and Western medicine treatments including lopinavir/ritonavir (Kaletra^®^), arbidol and Shufeng Jiedu Capsule (a traditional Chinese medicine) which is composed of mixture of flavonoids such as resveratrol and quercetin significantly improved pneumonia-associated symptoms in patients in the Shanghai Public Health Clinical Center, China [38,39]. Recently, siRNA-induced RNA interference (RNAi) play significant role and one of the fascinating techniques to explore the possible novel approach for emerging novel pathogenic viruses. For instance, siRNAs directed against Spike sequences and the 3′-UTR can inhibit the replication of SARS-CoV in Vero-E6 cells [40]. Abbott et al. demonstrated a CRISPR-Cas13-based strategy to inhibit SARS-CoV-2 inhibition by designing and screening of CRISPR RNAs (crRNAs) targeting conserved viral regions. The results revealed that they identified functional crRNAs targeting SARS-CoV-2 [41]. This technique could contribute immense level to inhibit spreading and infection of SARS-CoV-2. Taking all of this information into account, the increasing number of outbreaks and severity of viral infections call for novel, multidirectional, safe, biocompatible, cost-effective, target specific and tunable-based alternative approaches to prevent and treat the diseases caused by infectious viruses.

One such alternative approach involves nanomaterials and their fascinating properties, which include optimal size, shape, tunable surface charge, superparamagnetism, high surface plasmon resonance, luminescence, photon upconversion, bioavailability, biocompatibility, immunocompatibility/tolerability and biodegradability. Furthermore, the versatility of nanomaterials are can be easily decorated/anchored/conjugated with one or more type of functional groups, linkers and various bioactive molecules and some of the nanomaterials are being capable of simultaneous therapy and diagnosis [42,43,44,45].

In addition, the major requirements including cellular entry through the blood-brain barrier and blood-air barrier, tenability and targeted control discharge are feasible with nanomaterials, thus qualifying these materials as potential novel candidates for use in biomedical therapy [28,29,30] Nanoparticles have been widely used in antiviral therapy over the last few decades, owing to the development of surface functionalization strategies [45]. For example, Ag [46], Au [47], TiO_2_ [48], SiO_2_ [49], CeO_2_ [50] and CuCl_2_ [51] nanoparticles have been employed against different viruses including hepatitis B virus (HBV) [46], H3N2 and H1N1 [52], HIV-1 [53], herpes simplex virus (HSV) [54], vesicular stomatitis [55], foot-and-mouth disease [47] and dengue virus type-2 [51]. Recently, Sportelli et al. (2020) stated that researchers need to focus on the development of nanomaterial-based technological solutions to fight COVID-19 [56]. Therefore, several articles are expected to provide the basic knowledge regarding nanomaterials and describe how to use these materials for the development of antiviral therapies. Considering the seriousness of infectious disease transmission and the potential of nanomaterials in treating these diseases, our review first focuses on the mechanism of entry of viruses into host cells and then on the use of major and important types of nanomaterials such as silver, gold, quantum dots, organic nanoparticles, liposomes, dendrimers and polymers against various types of viral infections. Further, we discuss antiviral mechanisms, therapeutic approaches of nanoparticles and the effects of nanoparticles on CoVs. Finally, we provide our perspective on the potential of using nanoparticles in the future to treat infectious diseases.

## 2. Mechanism of Entry of Viruses into Host Cells

**Figure 1:**
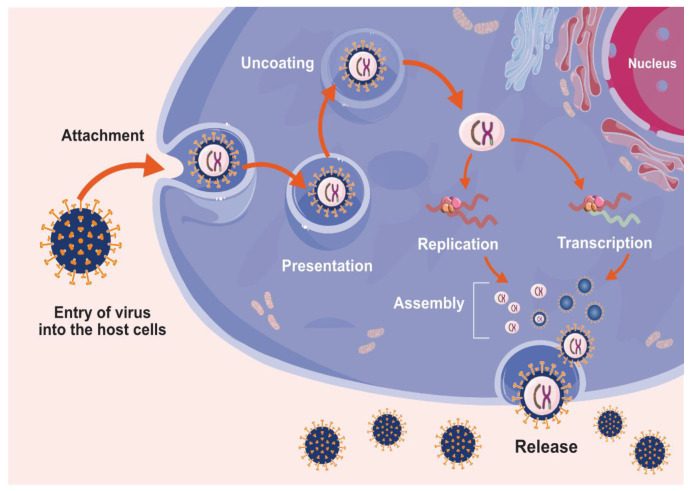
Mechanism of virus entry and replication in host cells.

Virus entry into host cells is required for viral multiplication. The infection process involves several steps including attachment, penetration, uncoating, replication, assembly and release (Figure 1). Viruses enter host cells through specific receptors on the host cell membrane using attachment proteins in the viral capsid or glycoproteins embedded in the viral envelope. The specificity of the interaction determines the kind of virus that infects the host cells. For example, bacteriophages enter the host cell through their nucleic acids and the capsid remains outside of the cell. Some animal and plant viruses enter host cells through endocytosis. Once inside the host cell, RNA viruses such as CoVs, use their genomic for the synthesis of viral genomic RNA as well as Mrna and eventually the release of the new virions produced in the host cells [57,58,59]. The human respiratory mucosa is the primary site of virus entry for various viruses including the influenza virus, respiratory syncytial virus and parainfluenza virus. These virulent pathogens primarily infect the upper respiratory tract but they may subsequently reach the lower respiratory regions causing more severe illness and ultimately morbidity and mortality. The clinical symptoms of the above pathogen attacks are frequently fever, dyspnea, cough, bronchiolitis and pneumonia [60]. Most respiratory tract infections are caused by SARS-CoV, MERS-CoV and SARS-CoV-2 [61]. A unique feature of CoVs such as SARS-CoV-2 is their mechanism of entry into host cells, initially through binding to the host-receptor cell at the angiotensin converting enzyme 2 (ACE2) protein site. This is followed by fusion with the host cellular membrane and subsequent release of the viral genetic material into the host cytoplasm or nucleus. The released viral RNA from CoVs is transcribed and the viral mRNA directs protein synthesis. Viruses replicate and assemble into new virions and these are released into neighboring cells via exocytosis. COVID-19 is caused by the novel CoV, SARS-CoV-2 [4,5]. SARS-CoV-2 is 80% and 50% homologous with SARS-CoV and MERS-CoV, respectively [4,62].

**Figure 2:**
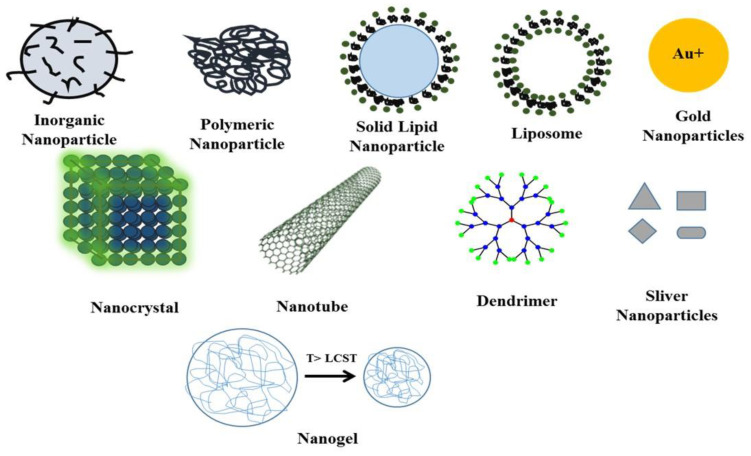
Various types of nanoparticles used for antiviral therapy as antiviral agents and delivery agents.

Viral diseases are major threat to human health and economy; therefore, it is necessary to find suitable, alternative, safe and biocompatible antiviral agents to prevent the spread of infections and reduce economic losses. Generally, nanoparticles including silver nanoparticles (AgNPs), gold NPs (AuNPs), quantum dots (QDs), carbon dots (CDots), graphene oxide (GO), silicon materials, polymeric NPs, dendrimers and polymers possess remarkable antimicrobial and antiviral activities [1,63,64,65,66,67]. Therefore, it is essential to highlight the variety and importance of selective nanoparticles that could be used as antiviral agents and delivery agents (Figure 2).

## 3. Silver Nanoparticles

Silver nanoparticles (AgNPs) are used as antiviral, antibacterial, anti-inflammatory, anti-angiogenesis, antiplatelet, antifungal and anticancer agents due to their unique physiochemical properties and superior biological functions [54,68,69]. Synthesis of AgNPs is carried out by various physical, chemical and biological methods. Biological methods appear to be environmentally friendly, safe, biocompatible and non-toxic. AgNPs have been used as biomedical therapeutic agents in wound dressings, long-term burn care products and anti-bacterial lotions [70]. Polyvinylpyrrolidone (PVP)-coated AgNPs homogenized in Replens gel (0.15 mg/mL) inhibited HIV-1 transmission of cell-associated and cell-free HIV-1 isolates after 1 min and offered long-lasting protection of cervical tissue from infection after 48 h treatment, with no evidence of cytotoxicity observed in the explants. AgNPs bind to glycoprotein gp120 on the HIV envelope in a manner that prevents CD4-dependent virion binding, fusion and infectivity [53]. AgNP-Coated polyurethane condoms (PUCs) efficiently inactivate HIV-1 and HSV-1/2 and their infectiousness; macrophage (M)-tropic and T lymphocyte (T)-tropic strains of HIV-1 are highly sensitive. The AgNP-coated PUCs can directly inactivate the microbe’s infectious ability and provide another line of defense against sexually transmitted microbial infections [71].

Fungi-Mediated synthesis of AgNPs reduced the viral infection dose in a size-dependent manner against HSV-1/2 and with human parainfluenza virus type 3 they blocked interaction of the virus with the cell, which might depend on the size and zeta potential of the AgNPs [72]. Antiviral activity of AgNPs and chitosan composites was evaluated against H1N1 influenza A virus. The composites showed significant antiviral activity in a size-dependent manner; surprisingly, chitosan alone did not show any antiviral effect. Conversely, AgNPs alone did exhibit antiviral activity; however, the composites showed remarkable antiviral activity compared to either AgNPs or chitosan alone [73]. AgNPs prevent transmissible gastroenteritis virus-induced apoptosis by regulating the p38/mitochondria-caspase-3 signaling pathway in swine testicle cells [74]. Curcumin-functionalized AgNPs demonstrated significant inhibitory effects against respiratory syncytial virus (RSV) infection by decreasing viral titers about two-orders of magnitude to non-toxic concentrations in host cells. Further, AgNPs prevented RSV from infecting host cells by inactivating the virus directly [75].

AgNPs showed antiviral and preventive effects against H3N2 influenza virus infection. In the presence of AgNPs, Madin-Darby canine kidney cells infected with H3N2 influenza virus showed better viability and no obvious cytopathic effects compared to an influenza virus control group. Infected mice treated with AgNPs showed lower lung viral titers and minor pathologic lesions in lung tissue and longevity [76]. Graphene oxide (GO)-AgNPs, composed of two nanomaterials in a single platform, were more effective than either single agent. GO-AgNPs inhibited feline CoV (FCoV) infection by 25% and infectious bursal disease virus (IBDV) infection by 23%, whereas GO alone only inhibited FCoV 16% and showed no antiviral activity against IBDV [77]. Huy et al reported antiviral activity of AgNPs against influenza A, HBV, human parainfluenza, HSV and HIV [78]. AgNPs synthesized using a green chemistry ultra-sonication approach exhibited antiviral activity against influenza A [79]. Zanamivir-loaded AgNPs synergistically inhibited H1N1 influenza virus multiplication [80]. Tannic acid-modified AgNP-based muco-adhesive hydrogel effectively reduced HSV-2 infectivity at the vaginal mucosal surface [81]. AgNPs exhibited antiviral activity against human oncogenic γ-herpesviruses, Kaposi’s sarcoma-associated herpesvirus and Epstein-Barr virus by reactivating viral lytic replication through the generation of reactive oxygen species (ROS) and autophagy [82]. Children are mostly affected by RSV; however, there is no specific treatment option available. The RSV virion contains two surface glycoproteins (F and G) that are vital for the initial phases of infection, making them critical targets for RSV treatment. AgNPs reduced RSV replication and the levels of pro-inflammatory cytokines (i.e., IL-1α, IL-6 and TNF-α) and pro-inflammatory chemokines (i.e., CCL2, CCL3, CCL5). Mice treated intravaginally with tannic acid (TA)-mediated AgNPs showed better clinical scores and lower virus titers in the vaginal tissues. The TA-mediated AgNP-treated group also showed significantly increased percentages of IFN-gamma+ CD8+ T-cells, activated B cells and plasma cells, while the spleens contained significantly higher percentages of IFN-gamma+ NK cells and effector-memory CD8+ T cells compared to NaCl-treated group. Further, the AgNP-treated animals showed significantly better sera titers of anti-HSV-2 neutralization antibodies than the NaCl-treated animals [83]. AgNPs interaction with HIV-1 in size-dependent manner and that the bound particles exhibit regular spatial relationships. AgNPs undergo preferential binding with the gp120 subunit of the viral envelope glycoprotein. These interaction between AgNPs and glycoproteins inhibit the virus from binding to host cells [84] and AgNPs act as potential antiviral agents against various type of viruses including influenza virus [85,86]. Biologically synthesized AgNPs using plant extracts of *Lampranthus coccineus* and *Malephora lutea* exhibited significant antiviral activity against different types of viruses such as HSV-1, HAV-10 and CoxB4 virus [87]. Altogether, all these studies demonstrated that the antiviral potential of AgNPs.

## 4. Gold Nanoparticles

Gold nanoparticles (AuNPs) have also drawn great interest in industry and nanomedicine due to their excellent electrical, optical, mechanical and biological properties [88,89]. AuNPs are used to detect DNA sequences, proteins, bacteria and viruses and they are frequently used in cancer studies and as antiviral and antibacterial agents. Gold nanorod, a GNR-5′PPP-ssRNA nanoplex-mediated immune activator, was reported to inhibit H1N1 influenza viral replication by upregulating the expression of IFN-β and other IFN-stimulated genes (ISGs), resulting in decreased viral replication [90]. Hyaluronic acid AuNPs and IFN complex have been used for targeted treatment of hepatitis C (HCV) infection [91] and highly mono-dispersed quasi spherical AuNPs inhibit HSV. Compared with the clinical drug acyclovir, AuNPs are very safe; they do not induce any drug-resistant viral strains and exhibit excellent viricidal properties [92]. Functionalized AuNPs suppress influenza virus, HSV and HIV. AuNPs potentially increase antiviral effects through multivalent interactions; dendronized AuNPs inhibit HIV more effectively than dendrons alone [93,94]. Sialic acid-functionalized AuNPs inhibit influenza virus infection, by multivalent interactions, relatively better than some synthetic clinical drugs such as zanamivir and oseltamivir, which are prone to resistance development by the influenza virus [95,96]. A study was performed to investigate the effect of AuNPs on Schistosoma mansoni-infected mouse liver. Comparing the treated and untreated infected groups, AuNPs significantly decreased the activities of malondialdehyde and nitric oxide and increased the level of glutathione (GSH). Concomitantly, AuNPs ameliorated the inflammatory response by decreasing the mRNA expression of interleukin (IL)-1β, IL-6, tumor necrosis factor (TNF)-α, IFN-γ and inducible nitric oxide synthase [97]. AuNPs also inhibited attachment and penetration of foot-and-mouth disease virus (FMDV) at the early stages of infection and impaired viral replication at later stages at non-toxic concentrations and at early stage [47]. AuNPs conjugated with peptide triazoles (AuNP-PT) exhibited significant antiviral effects against HIV-1 compared to the corresponding peptide triazoles alone. The enhanced virolytic activity and corresponding irreversible HIV-1 inactivation by AuNP-PT is due to multivalent contact between the nanoconjugates and metastable envelope spike (S) proteins on the HIV-1 virus [98]. Conjugation of M2e peptide with AuNPs and CpG as a soluble adjuvant (AuNP-M2e + sCpG) induced lung B cell activation and a robust serum anti-M2e immunoglobulin G (IgG) response in mice. Antibodies generated in response to the highly pathogenic avian influenza virus H5N1 (A/Vietnam/1203/2004) are capable of binding to the homotetrameric form of M2 expressed on infected cells [99]. Nanoparticles functionalized with the FluPep ligand showed enhanced antiviral activity compared to the free peptides. Conjugation of FluPep to AuNPs and AgNPs enhanced antiviral potency [100]. Gold nanoclusters (AuNCs), composed of tens and hundreds of gold atoms with an average size of 1–2 nm, play critical roles in biomedicine. Bai et al. (2018) reported that AuNCs prevented entry of porcine reproductive and respiratory syndrome virus (PRRSV) into host cells [101]. AuNCs selectively inhibited the proliferation and protein expression of PRRSV. Surface modification plays an important role in the antiviral activity of AuNCs; therefore, researchers modified the surface of AuNCs with two different materials—histidine and mercaptoethane sulfonate. Among these, histidine-stabilized AuNCs showed strong inhibition of the proliferation of pseudorabies virus (PRV) [101,102]. Therefore, AuNPs functionalized with different molecules play critical roles in antiviral activity.

## 5. Quantum Dots

Quantum dots (QDs) are nanocrystals of a semiconducting materials with an average size between 2–10 nm and these are widely used in cell labeling, detection and image tracking with particular size-dependent optical and electronic properties includes carbon, silver, gold, CdSeS/ZnS and so on. [103]. However, the uses of QD as an antiviral agent are very limited. Du et al developed GSH-capped cadmium telluride (Cd)Te QDs and demonstrated that they altered the structure of PRV surface proteins inhibiting the virus from entering the host cells [104]. Furthermore, the binding of CdTe QDs to the cell membrane itself also decreased viral numbers. Antiviral effects of stable carbon dots (CDots) with low toxicity were studied with PRV and PRRSV as test models of DNA and RNA viruses, respectively. CDots significantly inhibited the multiplication of both PRV and PRRSV. Furthermore, CDots remarkably induced the production of endogenous IFN and the expression of ISGs, which are responsible for virus replication [105,106]. The effect of various surface functionalizations of CDots was studied using 2,2′-(ethylenedioxy)bis(ethylamine) (EDA) and 3-ethoxypropylamine (EPA) against human norovirus virus-like-particles (VLPs), GI.1 and GII.4. Both EDA- and EPA-CDots (5 µg/mL each) were highly effective in inhibiting the binding of both strains of VLPs to histo-blood group antigen (HBGA) receptors on human cells. EDA-CDots achieved 100% inhibition and EPA CDots achieved 85–99% inhibition [107].

Benzoxazine monomer-derived CDots inhibited the infectivity of flaviviruses and non-enveloped viruses such as porcine parvovirus and adenovirus-associated virus by directly binding to the surface of virions and inhibiting the first step of virus and host cell interaction [108]. Cdots, surface-functionalized with boronic acid or an amine, inhibited the entry of HSV-1 into host cells by way of the alteration of viral coat proteins [109]. The use of biomolecules to prepare CDots provides stable and biocompatible materials. The antiviral effect of curcumin-stabilized cationic carbon dots (CCM-CDots) was evaluated against porcine epidemic diarrhea virus (PEDV) as a CoV model; CCM-CDots were found to inhibit the proliferation of PEDV with much higher efficiency than non-CCM-CDots [105]. CCM-CDots work by changing the viral surface protein structure, suppressing the synthesis of virus negative-strand RNA and virus budding and then inhibiting viral entry by generation of ROS, thus stimulating the production of ISGs and proinflammatory cytokines [105]. Carbon quantum dots (CQDs), derived from hydrothermal carbonization of ethylenediamine/citric acid, inhibited the entry and replication of HCoV-229E by interaction of the functional groups of the CQDs with HCoV-229E entry receptors on the host cell membrane [110]. Curcumin-mediated CQDs (Cur-CQDs) were prepared in a one-step heating process and injected with enterovirus 71 (EV71) in new-born mice. These Cur-CQDs exhibited superior antiviral effects [111]. Similarly, biocompatible CQDs prepared using glycyrrhizic acid (Gly-CDs) inhibited the proliferation of PRRSV by up to five-orders of viral titer [112]. Gly-CDs inhibited PRRSV invasion and replication, stimulated antiviral innate immune responses and suppressed the accumulation of intracellular ROS caused by PRRSV infection. Surface charge of the functionalized materials play critical role in the interactions between positively charged CDots and the negatively charged VLPs Positively charged EDA-CDots exhibited a higher inhibitory effect (~82%) than non-charged EPA-CDots (~60%) [107]. Both types of CDots also exhibited inhibitory effects on the binding of VLPs to their respective antibodies but they were much less effective in blocking the binding to HBGA receptors. After CDot treatment, VLPs remained intact and no degradation of the VLP capsid proteins was observed [107]. To summarize, the observed antiviral effects of CDots on noroviruses were mainly through effective inhibition of VLP binding to HBGA receptors and moderate inhibition of antibody binding, without affecting the integrity of the viral capsid protein and the viral particle. Collectively, QDs play critical roles in inhibiting various types of viruses.

## 6. Graphene Oxide

Graphene oxide (GO) is a unique single-atom-thick and two-dimensional carbon material arranged in a hexagonal lattice. GO and its derivatives are of immense interest to nanomedicine researchers due to their remarkable electronic, mechanical and thermal properties [113]. GO is widely used as an antibacterial and anticancer agent [114]. GO act as antiviral agent through inactivation of the pathogenic agent of hand-foot-and-mouth disease, EV71 and endemic gastrointestinal avian influenza A virus H9N2 [115]. Nanocomposites consisting of GO and partially reduced sulfonated GO (rGO-SO_3_) composite showed antiviral activity against HSV-1 by inhibiting HSV-1 from attaching to host cells [116]. The antiviral activity of GO and reduced GO was evaluated against PRV (a DNA virus) and PEDV (an RNA virus) revealing that GO significantly suppressed the infection of PRV and PEDV by a 2-log reduction in virus titers at non-cytotoxic concentrations. GO inhibited viral entry into the host cells by structural destruction [117]. Nanocomposites consisting of GO and AgNPs showed potential antiviral activity against enveloped and non-enveloped viruses, for example, FCoV and IBDV, respectively. Viral inhibition assays demonstrated that GO-AgNPs inhibited FCoV infection by 25% and IBDV by 23%, whereas GO alone only inhibited FCoV infection by 16% but showed no antiviral activity against IBDV infection. Therefore, the combination of GO and AgNPs exhibited better antiviral activity compared to either GO or silver alone [77]. Curcumin-Loaded GO exhibited a significant inhibitory effect on RSV infection with significant biocompatibility. GO inactivates directly by inhibiting the virus from attaching to host cells and considered to be coupled with prophylactic and therapeutic effects on the virus. The combination of GO and curcumin was more effective than either single agent against RSV infection [118]. Iannazzo et al. (2018) reported that the conjugation of GO and QDs (GQDs) potentially inhibited the replication of HIV [119]. Hypericin (HY)-loaded GO protected against novel duck reovirus (NDRV) disease, which is a serious infectious disease of poultry. The antiviral activity of the complex (GO/HY) was studied in DF-1 cells and in ducklings infected with NDRV TH11 strain. GO/HY showed a dose-dependent inhibition of NDRV replication, which may be attributed to direct virus inactivation or inhibition of virus attachment [120].

## 7. Zinc Oxide

Zinc oxide nanoparticles is a type of metal nanoparticles exhibited significant microbial activity against various type of microorganisms including viruses. The antiviral activity of zinc oxide (ZnO) micro-nano structures (MNSs) ZnO-MNSs was evaluated in virus infected corneal tissues. Partially negatively charged zinc oxide ZnO-MNSs efficiently trap the virions via a novel virostatic mechanism rendering them unable to enter into human corneal fibroblasts—a natural target cell for HSV-1 infection. Zinc oxide tetrapods (ZnOTs) significantly block the entry of Herpes simplex virus type-2 (HSV-2) into target cells and also stop the spread of the virus. The ZnOTs exhibit the antiviral activity by the ability to neutralize HSV-2 virions that natural target cells such as human vaginal epithelial and HeLa cells showed highly reduced infectivity when infected with HSV-2 virions [121]. Zinc oxide tetrapod nanoparticles (ZOTEN) induce immune system against HSV-2 virus and provide the therapeutic effects [122]. Zinc oxide tetrapods inhibit herpes simplex virus infection of cultured corneas [123]. The antiviral effect of zinc oxide nanoparticles (ZnO-NPs) and polyethylene glycol (PEG)-coated ZnO-NPs (ZnO-PEG-NPs) on herpes simplex virus type 1 (HSV-1). Zinc oxide nanoparticles (ZnO-NPs) and polyethylene glycol (PEG)-coated ZnO–NPs with concentration of 200 μg/ml inhibits at the rate of approximately 92% in copy number of HSV-1 genomic DNA and also it reduces virus titer [124]. Surface modified zinc oxide nanoparticles could modify the infection potential of HSV-1 via neutralizing the virus rather than through interfering with cellular targets by electrostatic interference of H-ZNPs with virus rather than the hydrophobic interaction ZNPs with virus [125]. Ghaffari et al. (2019) determined the antiviral activity of zinc oxide nanoparticles (ZnO-NPs) and PEGylated zinc oxide nanoparticles against H1N1 influenza virus [126]. The findings suggest that post-exposure of influenza virus with PEGylated ZnO-NPs and bare ZnO-NPs at the highest non-toxic concentrations could be led to 2.8 and 1.2 log10 TCID50 reduction in virus titer when compared to the virus control. The inhibition rate was much better in PEGylated ZNPs compared to unPEGylated ZnO-NPs. Zn^2+^ ions potentially inhibited Nidovirus replication and increasing concentration of the intracellular Zn^2+^ concentration can efficiently impair the replication of a variety of RNA viruses [127]. Prior incubation of HSV-2 with zinc oxide tetrapod nanoparticles (ZOTEN) inhibits the ability of the virus to infect vaginal tissue in female Balb/c mice and blocks virus shedding through neutralizing of virions particles in the host cells and also it stimulate a local immune response [128].

## 8. Organic Nanoparticles

Organic nanoparticles are widely used for drug delivery and therapeutic purposes in humans due to their biocompatibility, biodegradability and easy surface modification. The most common organic nanoparticles are polymeric nanoparticles, which are colloidal solids ranging from 10 to 1000 nm in diameter. The small size can facilitate capillary penetration and uptake by cells resulting in increased concentrations at target sites [129]. Polyhexylcyanoacrylate nanoparticles are loaded with either the HIV protease inhibitor saquinavir (Ro 31-8959) or the nucleoside analog zalcitabine (2′,3′-dideoxycytidine). Both nanoparticulate formulations led to a dose-dependent reduction of HIV-1 antigen production. Nanoparticles as a drug carrier system improved the delivery of antiviral agents to the mononuclear phagocyte system in vivo, overcoming pharmacokinetic problems and enhancing the activities of drugs for the treatment of HIV infection and AIDS [130]. Acyclovir loaded into beta-cyclodextrin-poly(4-acryloylmorpholine) (β-CD-PACM) nanoparticles exhibited significant antiviral activity against two clinical isolates of HSV-1 compared to both the free drug and a soluble β-CD-PACM complex [131]. 3D8 scFv-loaded PLGA (3D8-PLGA) NPs, potentially entered into the cytosolic regions of viral infected cells, were released continuously and hydrolyzed RNAs in the cytoplasm, thereby exhibiting maximal antiviral activity [132]. Diphyllin, a vacuolar ATPase blocker delivered by polymeric nanoparticles consisting of poly(ethylene glycol)-block-poly(lactide-co-glycolide) (PEG-PLGA), was more effective in inhibiting feline infectious peritonitis (FIP), caused by a mutated feline CoV, compared to diphyllin alone. Additionally, mice were more tolerant toward diphyllin-loaded nanoparticles. Therefore, nanoformulation with polymeric nanoparticles yielded potential antiviral activity against FIP [133].

The entry of viruses into host cells is complex and the process of interaction between the virus and host cell typically involves specific protein receptors. For example, multivalent flexible nanogels exhibited broad-spectrum antiviral activity by blocking virus entry and infection [134]. Previously, several studies used nanospheres for the treatment of HSV, HBV and influenza [135,136,137,138]. Altogether, organic nanoparticles can serve as drug delivery agents against various types of viral diseases.

Dendrimers are highly branched, symmetrical, macromolecular and hyper-branched structures radiating from a central core via connectors and branching units. Terminal groups are essential for targeting and interactions. Dendrimers are globular and contain three different regions—central core, branches and terminal functional groups. The potential functionality of dendrimers is due to encapsulation of several chemical moieties, interior layers and multiple surface groups [139]. In a study performed to determine the effect of various dendrimers on HSV-1/2, dendrimers BRI-2999 and BRI-6741 showed significant reduction of infection rates [140]. Two different type of peptide-derivatized dendrimers such as SB105 and SB105 A10 completely inhibited human cytomegalovirus (HCMV) replication and also inhibited murine CMV replication, whereas they were not able to inhibit adenovirus or vesicular stomatitis virus. The mechanism of inhibition of peptide-derivatized dendrimers namely SB105_A10 bound to human cells through an interaction with cell surface heparan sulfate and thereby blocked virion attachment to target cells [141]. Mice infected with Japanese encephalitis (JEV, GP78 strain) were administered with Morpholinos (5 mg/kg body weight) via intraperitoneal injection. Administration of Vivo-Morpholinos efficiently increased survival of animals and neuroprotection in a murine model of JEV [142]. SPL7013 is a dendrimer with broad-spectrum activity against HIV-1/2, HSV-1/2 and human papillomavirus. SPL7013 increased viricidal activity against HIV-1 strains that utilize the CXCR4 co-receptor [143]. Jyothi et al. (2015) developed novel liver-targeted cyclosporine A-encapsulated poly (lactic-co-glycolic) acid (PLGA) nanoparticles that inhibited the replication of HCV both in vitro and in vivo [144]. A liver-specific sustained drug delivery system, generated by conjugating a liver-targeting peptide to PEGylated CsA-encapsulated PLGA nanoparticles, reduced the immunosuppressive effects and toxicity profile of host factor cyclophilin A, which is essential for HCV replication [144].

To reduce the development of resistance by viral mutations, a study was performed with newly designed and efficient entry inhibitors. Antiviral activity dendrimers, as well as fullerene C_60_-with a unique symmetrical and 3D globular structure-were evaluated against an Ebola pseudotyped infection model. The central alkyne scaffold of fullerene connected to 12 sugar-containing [59] fullerene units (total 120 mannoses)-exhibited outstanding antiviral activity with an IC_50_ in the sub-nanomolar range [145]. The low concentration of camptothecin-loaded dendrimers inhibited HCV replication with very low toxicity. The triple combination of carbosilane dendrimers, tenofovir and maraviroc showed potential for inhibiting HIV sexual transmission [130,131,146]. Polyanionic dendrimers comprising the terminal groups sodium carboxylate, hydroxyl and succinamic acid and polycationic dendrimers containing primary amines were used to assess their antiviral activity in MERS-CoV plaque inhibition assays. The hydroxyl polyanionic set showed a 17.36–29.75% decrease in MERS-CoV plaque formation. All of these dendrimers showed excellent antiviral activity against MERS-CoV [147]. The unique properties of dendrimers are due to the presence of numerous surface functional groups, which facilitate conjugation with multiple drugs or targeting ligands. They also have the ability to encapsulate hydrophobic drugs due to their limited cavity size. VivaGel is a G4-poly-L-lysine dendrimer formed from the divalent benzhydylamine amide of L-lysine and it contains 32 terminal anionic functional groups used as an effective antiviral agent [148].

Polymers have high antiviral capacity due to their long chains and branches and their flexible molecular design. Polymers can be designed as arbitrary standards based on viricidal effects. They can be used not only as effective antiviral drugs but also as co-factors for treatment of viral infectious diseases. Polymers carrying antiviral drugs efficiently increase the solubility of antiviral drugs, thus prolonging the retention time and enhancing the uptake efficiency of drugs into cells. For example, organotin compounds were prepared according to the needs of universal viral agents [149]. Organotin and cisplatin-like polymers effectively kill viruses by inhibiting viral replication [150]. The polymers consist of poly(phenylene ethynylene) (PPE)-based cationic conjugated polyelectrolytes (CPE) and oligo-phenylene ethynylenes (OPE). They act as antiviral agents by the mechanism of oxidative stress, producing singlet oxygen species due to the π bonding system in the backbone of the compound upon exposure to UV-visible light. The oxidative stress induces damage to macromolecules including DNA, RNA and proteins [151]. Nucleic acid polymers containing hepatitis B surface antigen have been used to treat hepatitis D infection by binding to the amphipathic alpha helix in the class I surface glycoprotein [152]. Polymeric nanogels, which are cross-linked hydrogel particles comprising water-soluble and expandable polymers, are easily degradable into smaller sized fragments removed by renal clearance. These polymers can prevent the entry of virus particles into host cells by attaching to the heparan sulfate proteoglycans on the host cell surface. These flexible nanogels serve as robust inhibitors of HSV-2 virus infections [134]. Chun et al. (2018) designed amphiphilic copolymers comprising methoxy-poly(ethylene glycol)-block poly(phenylalanine), which consist of encapsulated mir-323a in the core and favipiravir in the exterior layer as both hydrophilic and hydrophobic antiviral agents [153]. These polymer-carried drugs serve to treat influenza A virus infectious diseases significantly better than naked drug delivery systems without polymers. The specific advantages of polymers are the sustained release of antiviral agents and the improved metabolic stability of the integrated drug. These properties demonstrate the great potential of polymeric particles for the successful delivery of antiviral agents [45]. Nanoviricide is a nanomachine that is armed to destroy a particular kind of virus using various types of nanoparticles. For example, polymeric nanoparticles were used to inhibit Varicella Zoster Virus infection in human skin and also polymeric nanoparticles were used for targeted drug delivery to prevent virus spreading and infections.

## 9. Liposomes

Liposomes are spherical in shape with an average size of 20 to 30 nm and are composed of a phospholipid bilayer containing an aqueous core [154]. Hydrophilic and lipophilic drugs can be incorporated into the inner aqueous cavity or the phospholipid bilayer, respectively. Liposomes are non-toxic and biocompatible. When the HIV virus envelop region T cell receptor targeted antisense RNA was encapsulated in liposomes, HIV-1 production was completely inhibited [155]. Hematopoietic toxicity and antiviral activity of liposomal-encapsulated 3′-azido-3′-deoxythymidine (AZT) were examined in mice for 5 days following intravenous administration (0.4 to 10 mg/kg body weight); a significantly depressed bone marrow cellularity as well as a corresponding decrease in red blood cells, blood neutrophils and monocyte numbers were observed. Treatment with liposomal AZT significantly reduced virus proliferation using AZT alone [156]. Nanocarbon fullerene lipidosome (NCFL) inhibited influenza virus H1N1 by a direct killing effect, which was comparable to that of rimantadine hydrochloride [157]. To develop broad-spectrum antiviral agents against a variety of viral infections, polyunsaturated liposomes (PLs) were fabricated for use in in vivo drug delivery and to specifically target the endoplasmic reticulum. These PLs contained polyunsaturated fatty acids that exhibit independent antiviral activity by reducing cellular cholesterol. Targeting cholesterol biosynthesis within infected cells is one of the promising targets of many viral systems, including HCV, HBV and HIV [158]. PLs significantly decreased viral infectivity and secretion in HCV, HBV and HIV infections. Pretreatment of cells with PLs reduced the infectivity of both HCV and HIV by suppressing plasma membrane cholesterol levels. Cationic liposomes, containing both a fluorescence marker (calcein) and antiviral drugs HPMPC (Cidofovir^®^), were internalized in MDBK cells infected with bovine HSV-1 and significantly inhibited viral replication [159]. Norovirus RdRp is used as an antiviral agent and it is a promising target enzyme for the development of new antiviral drugs. The polysulfonated naphthylurea suramin has the potential to inhibit murine and human norovirus polymerases. Suramin-loaded liposomes exhibited significant antiviral activity against murine norovirus cultivated in RAW 264.7 macrophages [160]. Furthermore, surmamin inhibited the replication of various chikungunya viral (CHIKV) isolates including Sindbis virus and Semliki Forest virus. Various studies revealed that the antiviral activity and mechanism of suramin is through interference with (re)initiation of RNA synthesis. The antiviral effect of suramin-containing liposomes potentially prevents and helps treat CHIKV infections [161]. Acyclovir-Loaded flexible membrane vesicles showed significant antiviral activity in a murine model of cutaneous HSV-1 infection. The lipid based system exhibited safe treatment [162]. Cationic liposomes loaded with stearylamine (SA) inhibited viral infectivity without preloaded active pharmaceutical ingredients. SA liposomes suppressed baculoviral infectivity in several mammalian cell lines, including A549 cells. The SA liposomes are non-toxic and could increase antiviral effects by reducing cholesterol content, which intensify concurrently with increased binding of SA liposomes to the cell membrane. SA liposomes potentiate the entry of virus particles into host cells and are compatible with the antiviral drug acyclovir [163]. Hence, liposomes are the best carrier molecules to deliver antiviral drugs.

## 10. Antiviral Mechanism of Nanoparticles

**Figure 3:**
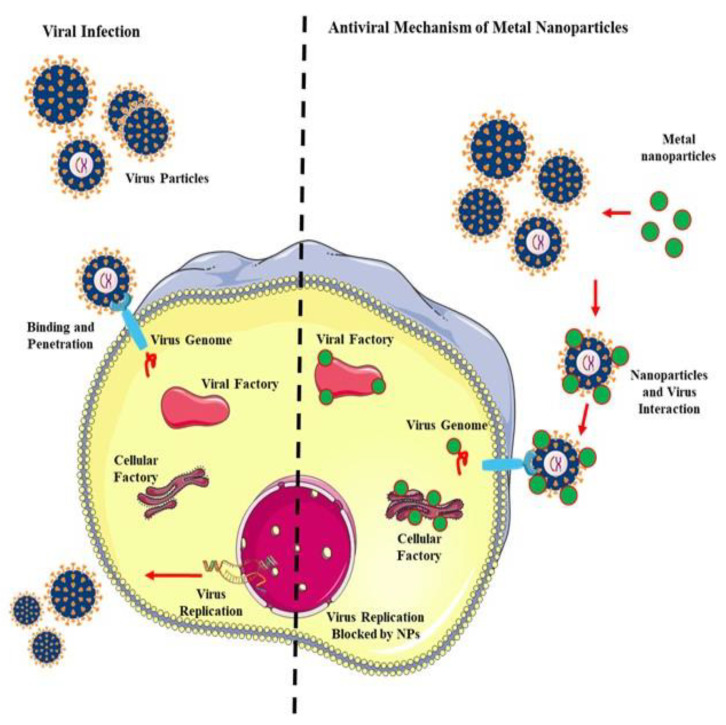
Antiviral mechanism of nanoparticles (NPs).

Antiviral mechanisms of nanoparticles should target attachment, penetration, replication and budding of viruses. Possible mechanisms involve inactivation of the virus directly or indirectly, prevention of attachment of viruses to host cells and blocking viral replication but they also depend on the form and type of nanoparticles used [57]. Most often, nanoparticles block the above steps by altering the structure of the capsid protein and eventually reducing virulence, which can be attributed to both physical and chemical means of decreasing the active viral load. For example, Lara et al. (2010) demonstrated that AgNPs bind to glycoprotein gp120 of the HIV envelope preventing CD4-dependent virion binding, fusion and infectivity in cell-free and cell-associated viral assays [53,164]. Recently, Cagno et al demonstrated the antiviral mechanisms of broad-spectrum, non-toxic nanoparticles against HSV, human papilloma virus, RSV, dengue and lenti virus [165]. The authors showed that a series of antiviral nanoparticles with long and flexible linkers mimicking heparin sulfate proteoglycans, the highly conserved target of viral attachment ligands (VALs), could achieve efficient viral prevention. This was through effective viral association with binding simulated to be strong and multivalent to the VAL repeating units [165]. Water-Soluble fullerene-polyglycerol sulfates prevented interaction of vesicular stomatitis virus coat glycoprotein with baby hamster kidney cells [166]. Lysenko et al. (2018) proposed that one of the main and direct mechanisms of nanoparticle-mediated antiviral activity is linked to local-field action against the receptors at the virus surface [50]. In this process, the nanoparticles adsorbed on the surface of the cell can greatly alter the membrane potential. On the other hand, the indirect antiviral mechanism of nanoparticles includes blocking the penetration of the virus into the cell due to a change in membrane potential. Collectively, these reports suggest that the main mechanisms of nanoparticle antiviral activity involve interaction with gp120, competitive binding between the virus and nanoparticles for the host cell, interference with viral attachment, inhibition of virus-host cell binding and penetration, binding to the plasma membrane, inactivation of viral particles prior to entry and interaction with double-stranded DNA and/or binding with viral particles [96,135,164,167,168,169,170]. While metal nanoparticles interact with viral particles, replication of the virus is blocked (Figure 3).

Recently, Cagno et al. [165] demonstrated the state of the art mechanism of non-toxic nanoparticles against various type of viruses. The authors developed antiviral products, which usually mimic heparan sulfate proteoglycans (HSPG), as well-preserved target of “viral attachment ligands” (VALs). The antiviral effect relies on the binding mechanism of the nanoparticles to the virus surface, thus preventing virus-cell attachment. The most outstanding viricidal effect was found in the AuNPs coated with a 2:1 mixture of decanesulfonic acid (MUS) and 1-octanethiol (OT). MUS allows a multivalent binding as a consequence of its structure comprising a long hydrophobic chain, sulfonic acid terminated. The enhanced activity of MUS:OT-NPs was assigned to the new construct using MUS linker that caused local distortions and then a global virus deformation, leading to irreversible loss of infectivity. The MUS:OT-NPs exhibited efficient virucidal effect against HSV-1 and HSV-2, human papilloma virus (HPV-16), respiratory syncytial virus (RSV), dengue and lenti virus. The synthesized MUS:OT-NPs exhibited toxic to the virus and non-toxic to the host cells [165].

The possible mechanism of antiviral activity of non-toxic nanoparticles such as gold inhibits viral replication and prevent release of viral particles into the host cells. The inhibition can take place by nanoparticles act as blockers of neuraminidase enzyme which cleaves the attachment between hemagglutinin on the progeny virus and sialic acid receptor on the host cell. In this case, nanoparticles prevent this cleavage step and interfere with the release of progeny virus from infected host cells and subsequently, prevent the progression of infection. Therefore, the possible antiviral mechanism could be inhibition of hemagglutinin and neuraminidase activities. In addition, like gold nanoparticles blocking attachment of virus into the host cells.

## 11. Effects of Nanoparticles on Coronaviruses

Over the last decade, nanoscience and nanotechnology have played a critical role in nanomedicine due to the size, shape and surface charge of nanoparticles as carriers, detection agents and direct inhibitory agents against microbes and cancer cells. Nanoparticles have been shown to be efficient and selective agents for the delivery of therapeutic moieties such as drugs, vaccines, siRNAs and peptides. In addition, nanoparticles have been used to detect and monitor diseases and treat responses using noninvasive imaging modalities. The size of nanomaterials facilitates their involvement in the efficient delivery of antigens due to surface functionalization; they have the capability of co-transporting antigens accompanied by numerous adjuvants. Nanomaterials are able to deliver the drug at suitable concentrations in a precise manner, to the proper place and at the proper time [171].

**Figure 4:**
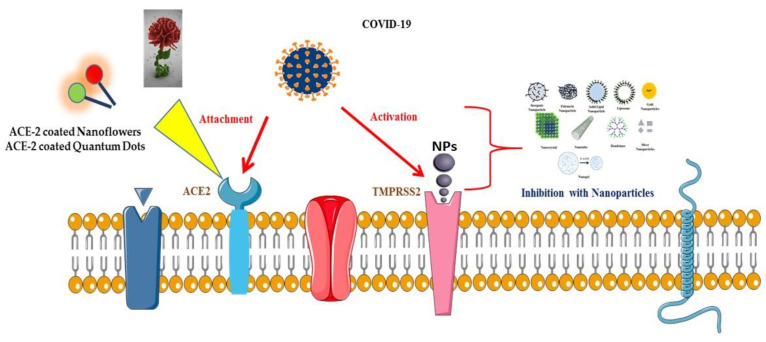
Hypothetical model illustrating the entry of SARS-CoV-2 into a host cell and its pathogenicity. SARS-CoV-2 spike (S) proteins bind to ACE2 receptors and then the virus enters and infects host cells. ACE2 activation may be blocked by ACE2-coated nanoflowers and ACE2-coated quantum dots. Nanoparticles (NPs) may prevent or reduce the activation/expression of the cellular protease TMPRSS2, which primes the S proteins, potentially suppressing SARS-CoV-2 infection.

An important aspect of nanomaterials in relation to CoVs, is that they are able to inhibit or compete with viral binding to the host cell-surface receptor. For example, the ACE2 receptor plays a critical role in the entry of CoVs into host cells, especially in the case of SARS-CoV and SARS-CoV-2 [172]. Hence, blocking and/or decreasing the levels of ACE2 could help in the fight against infection, as well as in the development of antibodies against ACE2. On the other hand, ACE2 has a protective effect against virus-induced lung injury after infection as a result of increasing the production of the vasodilator angiotensin 1–7 [173]. Therefore, preventing COVID-19 in the host can be more effective than fighting against the virus after infection. Instead of developing vaccines, which typically takes a long time and several rounds of protocols and trials, it would be better to try to prevent entry of these viruses into humans using nanotechnology for preparing masks, clothes, gloves and gums by exploiting ACE2-coated nanoflowers and QDs [174]. In addition, researchers have demonstrated that nanoflowers and ACE2-coated QDs can be used as bio-detection probes. Furthermore, they have been used to enhance catalytic activity and stability of ACE2 and also to produce chewing gums, nose filters, masks, clothes and gloves to prevent entry and spread of CoVs [174,175]. Therefore, if materials used in hospitals were prepared with coated nanoparticles, the spread of CoV infection may be inhibited. Herein, we propose a hypothetical antiviral mechanism involving ACE2-coated nanoflowers and ACE2-coated QDs that could block SARS-CoV-2 entry into cells by inhibiting attachment of the virus to the ACE2 protein, while also inhibiting activation of the accessory serine protease TMPRSS2 (Figure 4).

A nanoparticle-based intranasal delivery system is an effective and safe tool to deliver several therapeutic moieties such as vaccines, drugs, siRNAs, peptides and antibodies. Intranasal delivery is noninvasive, practical, simple and cost effective. The intranasal delivery system has been evaluated for vaccination against respiratory viruses such as influenza and CoVs [176,177]. Nanoparticle-Mediated delivery systems have the following benefits—free from enzyme degradation; long-term existence, release and retention within the system; amicable co-delivery with adjuvants; specific targeting of cells through receptor-ligand interactions; and potentiation of the immune system [178,179].

There are major challenges in combatting infectious diseases such as SARS, MERS and COVID-19, including the fact that there are no effective drugs or vaccines available. Bachmann and Jennings (2010) reported that nanoparticles have the potential to enhance transport in the lymphatic system compared to smaller subunit antigens. Virus-Like nanoparticles (VLNPs) play a significant role in vaccine development as vaccine carriers and they can stimulate host-immune responses [180]. Nanoparticle-based vaccines have shown much promise in improving vaccine efficacy, immunization strategies and targeted delivery. VLNPs improve vaccine efficacy, protect the antigens from premature proteolytic degradation, facilitate antigen uptake, control release and they are non-toxic [181]. VLNPs are composed of a self-assembled viral membrane that forms a monomeric complex displaying a high density of epitopes [182]. VLNPs can accommodate the expression of additional proteins or the endogenous expression of multiple antigens [183,184]. Due to these specific features and unique qualities, VLNPs can provide protection not only against viruses but also against heterologous antigens [185]. A host-immune response was generated after the delivery of an antigen using virus capsid protein SV40 in mammalian cells [186]. VLNPs can increase the immunogenicity of weak antigens including *Salmonella typhi* membrane antigen and influenza A M2 protein. VLNPs containing H1V1 Nef gonadotropin releasing hormone (GnRH) assembled and produced a strong antigen-specific humoral response as well as a cellular immune response [187,188]. VLNPs seem to be better carrier molecules, owing to their multiple surface antigens, compared to antigen presenting cells, which can only present one type of antigen on their surface [189]. Another interesting feature of VLNPs is their high surface energy, which leads to strong adhesion of biomolecules. These qualities contribute to a virus mimicking effect that stimulates the immune system to produce antibodies and immune cells to fight viral infections [190,191]. The combination of AuNPs (with an average size of 100 nm) and spike (S) proteins of infectious bronchitis virus exhibited increased stability when used to developed VLNPs and showed a significant retention of the S proteins compared to viral antigens [192]. VLNPs mediated the inhibitory effects of MERS-CoV S protein nanoparticle vaccine and matrix (M1) protein adjuvant combination on MERS-CoV replication in the lungs of mice. In addition, the MERS-CoV S nanoparticle vaccine produced a high titer of anti-S neutralizing antibodies and protected against MERS-CoV infection in mice in vivo. Altogether, these studies suggest that VLNPs conjugated with S protein seem to be a potential design for a successful vaccine, not only to stimulate the immune system but also to protect humans from MERS-CoV. This approach can also be applied to SARS-CoV-2 as both CoVs use the same mechanism of entry into host cells and similar pathogenicity [193]. Taken together, nanoparticles can be used as antiviral agents against various types of viruses including SARS-CoV-2.

## 12. Therapeutic Approaches for Coronaviruses

Emerging and reemerging viruses are responsible for a number of recent epidemic outbreaks. A vital part of controlling of viruses are predicting and controlling of spreading and infections. Although we are developing various kinds of antiviral drugs to stop spreading and infections of deadly viruses, the detection of pathogen is very critical in the first place. Therefore, researchers are interested to develop sensitive, rapid, simple technique for accurate characterization of emerging virus strains. Manual magnetic particle-based extraction methods were developed to detect HIV and HCV viral nucleic acids combination with detection by reverse transcriptase-polymerase chain reaction (RT-PCR) one-step. These methods can be used to routinely screen blood donation for viremic donors [194]. Liu et al. (2013) developed a rapid diagnostic platform for pathogen detection based on the acetylcholinesterase-catalyzed hydrolysis reaction which is comparable to that of PCR and easily detectable through changing of color [195]. Silica-Coated magnetic nanoparticles were prepared by co-precipitation method. These Fe_3_O_4_/SiO_2_ nanoparticles were used to isolate genomic DNA of hepatitis virus type B (HBV) and of Epstein-Barr virus (EBV) for detection of the viruses based on polymerase chain reaction (PCR). The results depicted that the purification efficiency of DNA of both HBV and EBV using obtained Fe_3_O_4_/SiO_2_ nanoparticles was significantly better that commercially available reagents [196]. Yeh et al. (2020) developed a portable microfluidic platform containing carbon nanotube arrays with differential filtration porosity for the rapid enrichment and optical identification of viruses [197]. This technique used to characterize various type of viruses including rhinovirus, influenza virus and parainfluenza viruses. This enrichment method could be used to rapidly track and monitor viral outbreaks in real time. Zhao et al. (2020) developed efficient magnetic nanoparticles-based viral RNA extraction method to detect SARS-CoV-2, which is comparable with PCR techniques [198].

Recently, SARS-CoV-2 emerged as a global threat for both healthcare and the economy. Currently, a variety of antiviral agents, including re-purposed drugs, are under testing in clinical trials to assess their efficacy against this new virus but the mission of finding an effective treatment for COVID-19 is ongoing [199,200,201,202]. Previously, several modes of treatment were practiced against MERS and SARS infections including the use of inhibitors of viral and host proteases, IFNs and host-directed therapies. Ribavirin, a nucleoside analog that acts as an RNA polymerase inhibitor was used on patients with SARS and MERS [203,204]. Development of new therapies should focus on the CoV S protein because it guides the entry of CoVs into host cells by participating in the binding and fusion of the virus to the ACE2 receptor on the host cell membrane. The S protein is composed of two subunits—S1 recognizes and binds to host receptors and S2 facilitates fusion between the viral envelope and the host cell membrane [205]. Although several agents have been developed using peptide fusion inhibitors, anti-CoV neutralizing monoclonal antibodies and entry receptor antagonists, none of these potentially curative agents is approved for commercial use in humans [206]. Remdesivir, an adenosine analogue, is a broad-spectrum antiviral with potent in vitro efficacy against multiple genetically-unrelated RNA viruses including Ebola, SARS-CoV and MERS-CoV [38]. Remdesivir seems to be an effective antiviral agent against COVID-19 [207]. Another drug, chloroquine, has an immunomodulatory effect and functions at both the entry- and post-entry stages of SARS-CoV-2 infection [208]. However, all of these agents have undesired side effects. Therefore, other agents such as nanoparticles need to be explored as alternative antiviral agents.

Recently, nano and nanomediated combination therapy (nanoparticles plus antiviral drugs) have shown immense promise in nanomedicine. Metal nanoparticles and metal-loaded nanocomposites are known to be extremely effective against microbes and viruses due to their unique property, the controlled release of ions. For example, the controlled release of ionic copper is the fundamental aspect for the antimicrobial and antiviral properties of surfaces [209]. In addition, the controlled release of ions favors the production of ROS. Metal-grafted GO decorated with metals such as Ag, Fe, Cu, Zn, TiO2, CdS and MnS_2_ exhibited potential antiviral activity [77,210]. For example, silver and copper decorated GOs are potential antiviral agents for both enveloped and non-enveloped viruses [77]. The development of nanomaterial-mediated therapy is an alternative to conventional therapies in fighting against resistant viruses [211]. AgNPs are able to interact with host receptors and inhibit viral entry. Functionalized AuNPs showed antiviral activity against HIV-1 and also against H1N1, H3N2 and H5N1 [211].

Adaptor protein complex 2 (AP2)-associated protein kinase 1 (AAK1) is a key regulator of endocytosis. Hence, a drug such as baricitinib that inhibits AAK1 may suppress viral entry into target cells. As such, baricitinib could be a potential treatment for COVID-19 [212]. Baricitinib has been shown to bind to another endocytosis regulator, cyclin G-associated kinase and inhibit AAK1, thus preventing viral entry into the cell [213]. HIV protease inhibitors such as lopinavir and ritonavirin suppress 3-chymotrypsin-like protease activity of SARS-CoV and MERS-CoV [214]. Remdesivir, a nucleoside analogue that targets RdRp, suppresses viral RNA synthesis in a broad spectrum of RNA viruses including HCoVs. Remdesivir inhibited RdRp of CoVs in cell cultures and animal models [201,215]. A combination of hydroxychloroquine and azithromycin can potentially increase the recovery of COVID-19 patients. Chloroquine-Based drugs inhibit the fusion of SARS-CoV-2 with host cells by acidifying the lysosomes and thus inhibiting cathepsins that require a low pH for optimal cleavage of SARS-CoV-2 S protein. These drugs can also alter cross-talk between SARS-CoV-2 and host cells and reduce production of pro-inflammatory cytokines, while activating anti-SARS-CoV-2 CD8+ T-cells [216,217].

## 13. Conclusions and Future Perspectives

Infectious diseases cause immense global mortality and viruses are responsible for about one-third of these deaths. Respiratory infections are among the most common causes of death worldwide, especially due to CoVs. Presently, the outbreak of viral respiratory infections, particularly COVID-19, is widespread and continuing to spread worldwide. As of 27 July 2020, SARS-CoV-2, the infectious agent of COVID-19, had infected 16,430,566 individuals and led to the death of more than 652,434 individuals in 215 countries, while also triggering an exceptional economic crisis. Although there are no specific drugs or vaccines to treat or prevent COVID-19, the available antiviral drugs are re-purposed drugs and active against a limited panel of human pathogens. Therefore, researchers are urgently striving to identify and develop suitable nano-based drugs and nano-vaccines, in addition to conventional approaches. Nanotechnology plays a critical role in both viral disease diagnosis and therapeutics. Nanoparticles show great potential for biomedical applications, especially in patients who relapse after completing conventional antiviral therapy. Antiviral resistance, which is a slowly developing problem of conventional therapeutic approaches, may be addressed using nanoparticles due to their large surface-to-volume ratio, surface charge, size and shape as well as their optical, electronic, biological and functional properties. Furthermore, nano-based approaches are feasible, cost effective, non-toxic, biocompatible and a convenient strategy to deal with various types of viral infections, particularly SARS-CoV-2/COVID-19. In this review, we have provided a brief account of the main mechanism of entry of viruses into host cells. We also discussed in detail the effects of several important types of nanomaterials including AgNPs, AuNPs, QDs, organic nanoparticles, liposomes, dendrimers and polymers against various types of viral infections. These functional nanoparticles can provide a novel platform for fabrication of bio-safe and effective drugs for nanoscale treatment of viral infectious diseases. Further, we discussed antiviral mechanisms, therapeutic approaches of nanoparticles and the effects of nanoparticles on CoVs. Finally, we provide our future perspective of nanoparticles below.

Nanotechnology has become a focal point of research and various types of nanomaterials have been explored and evaluated for their prophylactic and therapeutic activity against different viruses. Therefore, nanoparticle-based therapy seems to be promising but there are still many challenges and barriers to achieve its full potential. The future focus of antiviral nano-based therapy should concentrate on development of new antiviral therapeutics and approaches to challenge the emergence of drug resistance and different secondary effects due to long-term conventional treatments. Antiviral drugs currently available are effective against a few viral diseases such as influenza, hepatitis, HSV and HIV. Generally, development of an antiviral drug takes a long time (years) and the process involves tedious protocols, particularly for CoVs due to their variability. Most of the antiviral drugs developed through nanoparticle research show immense potential in in vitro and in vivo conditions; however, several variables need to be optimized for a successful translation of nanomaterials from the laboratory to the clinical setting. In addition, one important aspect is the non-toxic nature of nanoparticles, which require long and intense studies to demonstrate their non-toxic nature, let alone their potential activity against specific viruses. The biotech/nanotech industry needs more exposure to demonstrate the effects of nanoparticles on health and their applications in various fields. The crucial points and future focus of nano-based antiviral drugs need to be on the following important issues. First, the system needs to have receptor-based nanoparticles, which could be safely managed as antiviral agents to rapidly target specific viruses. Second, functionalization by specific molecules is needed to facilitate effective targeting of specific sites of viral infections. Third, non- or reduced-toxicity must remain a priority; biocompatibility with no undesired side effects is essential. Fourth, a therapeutic approach that combines nanoparticles and low concentrations of antiviral drugs with excellent efficiency, is needed. Finally, a multidisciplinary consortium is needed to address potential questions related to various types of viruses, variability, frequent mutations and antiviral agents and their use in humans, particularly during pandemic situations as we are now experiencing. We believe that the approach presented here has a chance to produce medically-relevant antiviral drugs against CoVs and other viral diseases.
